# Multichromophoric sugar for fluorescence photoswitching

**DOI:** 10.3762/bjoc.10.151

**Published:** 2014-06-30

**Authors:** Stéphane Maisonneuve, Rémi Métivier, Pei Yu, Keitaro Nakatani, Juan Xie

**Affiliations:** 1 PPSM, ENS Cachan, CNRS, UMR8531, 61 avenue du Président Wilson, 94235 Cachan cedex, France; 2 LCI, ICMMO, CNRS, Université Paris-Sud, 15 rue Georges Clémenceau, 91405 Orsay Cedex, France

**Keywords:** click chemistry, energy transfer, fluorophore, monosaccharide, photochromism

## Abstract

A multichromophoric glucopyranoside **2** bearing three dicyanomethylenepyran (DCM) fluorophores and one diarylethene (DAE) photochrome has been prepared by Cu(I)-catalyzed alkyne–azide cycloaddition reaction. The fluorescence of **2** was switched off upon UV irradiation, in proportion with the open to closed form (OF to CF) conversion extent of the DAE moiety. A nearly 100% Förster-type resonance energy transfer (FRET) from all three DCM moieties to a single DAE (in its CF) moiety was achieved. Upon visible irradiation, the initial fluorescence intensity was recovered. The observed photoswiching is reversible, with excellent photo resistance.

## Introduction

**Figure 1:**
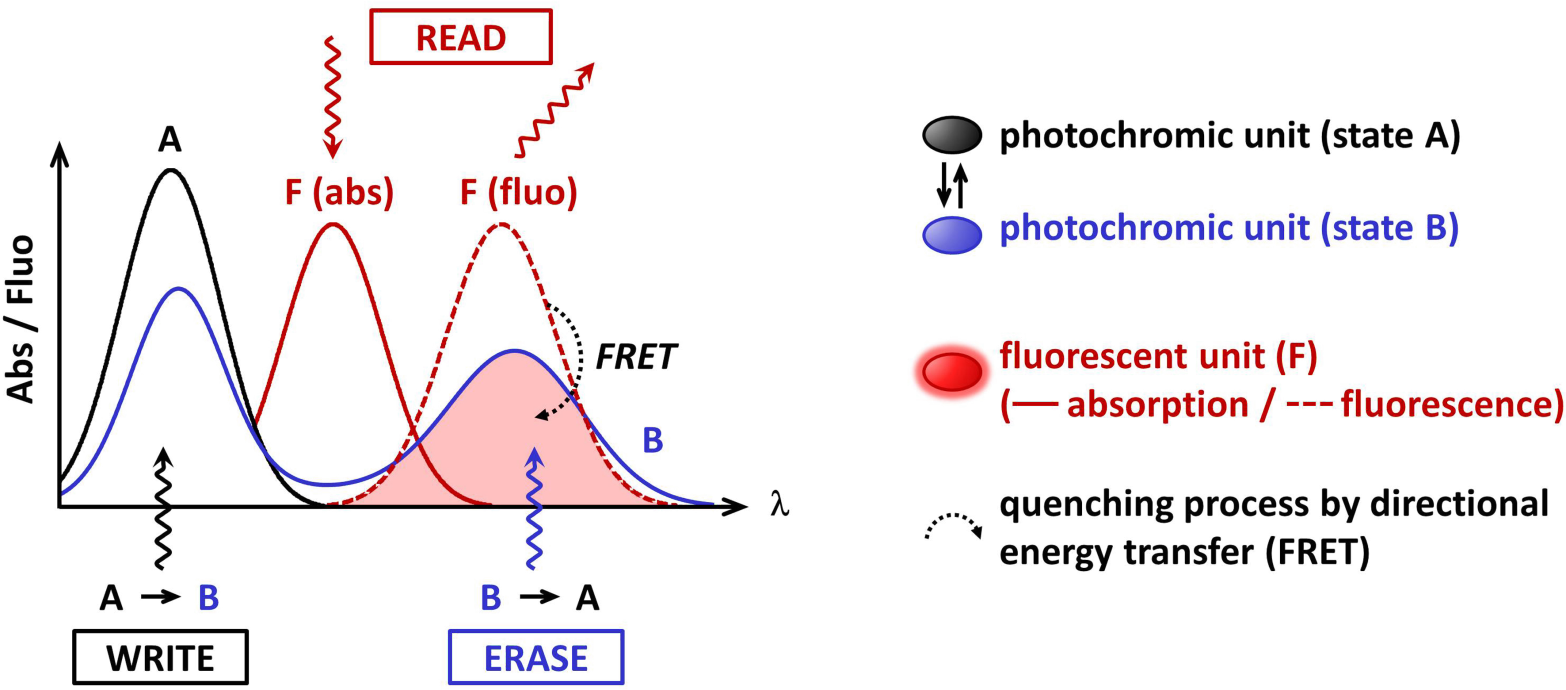
Requirements on absorption and emission spectral features of the photochromic and fluorescent units of the platform to induce efficient fluorescence photoswitching by energy transfer process (FRET).

The development of functional nanomaterials is nowadays a very attractive field of fundamental and applied research. The chemical functions at the molecular level yield properties, which are translated in terms of specific electronic or optical functions to the materials and device level. One of the challenges is to synthesize organic nano-architectures with a high degree of structural order and well-defined properties yielding high-performance functions, for both economic and environmental saving reasons. Saccharides are polyfunctional molecules with well-defined stereogenic centres in one molecular unit, and constitute “platforms” on which multiple functional moieties can be attached. Among many applications, the use of photochemical devices based on sugar derivatives is particularly appealing for the development of supramolecular systems for optical data storage media. Photochromic molecules are particularly efficient photo-driven switches, as they can commute upon light excitation between two distinct molecular species (states A and B, Figure 1) with the possibility to cycle up to one million “round trips”, showing different physical and chemical properties, the most noticeable one being the absorption change [1]. Indeed, they usually shuttle between a colorless and a colored form. Combining them with fluorescent compounds provides an added value to their photophysical function. Actually, fluorescence allows the possibility to reach high sensitivity and very low detection levels, down to the single molecule limit, whereas absorption spectroscopy requires a high number of active molecules [2]. If structural and spectral features of the fluorophore and the photochromic compound fit well together, the fluorescence can be switched ON and OFF: quenching of the fluorescence through a Förster-type resonance energy transfer (FRET) process from the former to the latter would occur, when the photochromic moiety is in the colored form (state B, Figure 1). At the opposite, in the colorless form (state A), the absence of FRET would keep the fluorescence alive, showing that the combination of these two functional molecules leads to a photon-driven fluorescence switch.

**Figure 2:**
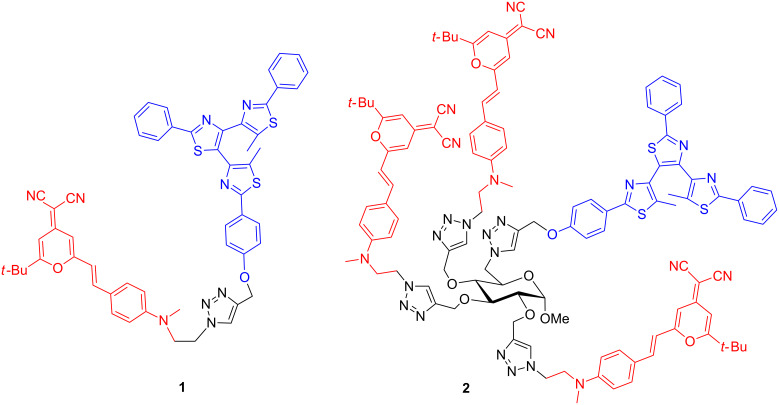
Bifunctional fluorescent-photochromic molecules **1** and **2**.

Previously, we have synthesized a fluorescent-photochromic dyad (**1**, Figure 2) combining a DCM fluorophore (4-dicyanomethylene-2-*tert*-butyl-6-(*p*-dialkylaminostyryl)-4*H*-pyran) [3] with a photochromic diarylethene (DAE) which showed a photoreversible two-way FRET controlled by the state of the photochromic moiety, and 49% quenching of the fluorescence upon UV irradiation [4]. Bifunctional molecules built on two distinct (photochrome and fluorophore) moieties have also been reported by other groups [5–9]. The fluorophore vs photochrome ratio in reported bifunctional systems is 1:1 [4–7] or 2:1 [8–9]. Other strategies to assemble interacting fluorophores and photochromes (supramolecular systems, nanoparticles, polymer materials) are also reported [10–13]. FRET operates at distances of a few nanometers around the photochromic moiety (in its state B, Figure 1), which is related to the Förster radius. It means that in a suitably designed molecular system, one single photochromic unit can quench several fluorophores present within this distance. In a situation where one photochromic unit is surrounded by several fluorophores, we can take advantage of this phenomenon both to increase the brightness of the fluorescent molecular system and to turn “ON” and “OFF” several fluorophores with one given photochromic molecule. This is valuable in terms of photon (thus energy) saving, since “switching a few photochromes leads to quenching many fluorophores”. In this perspective, we decided to design a multichromophoric glycopyranoside bearing three DCM fluorophores and one photochromic bis(dithiazole)ethane [14–16] (compound **2**, Figure 2) so as to take advantage of this sugar-based “platform” to get a specific molecular architecture and to study the energy transfer and photo-switching efficiency. To the best of our knowledge, readily available monosaccharides have been rarely used to develop multichromophoric supramolecular systems. Only one artificial light-harvesting antenna system grafted on the α-D-glucopyranoside has been reported [17].

## Results and Discussion

### Synthesis of multichromophoric sugars

**Scheme 1:**
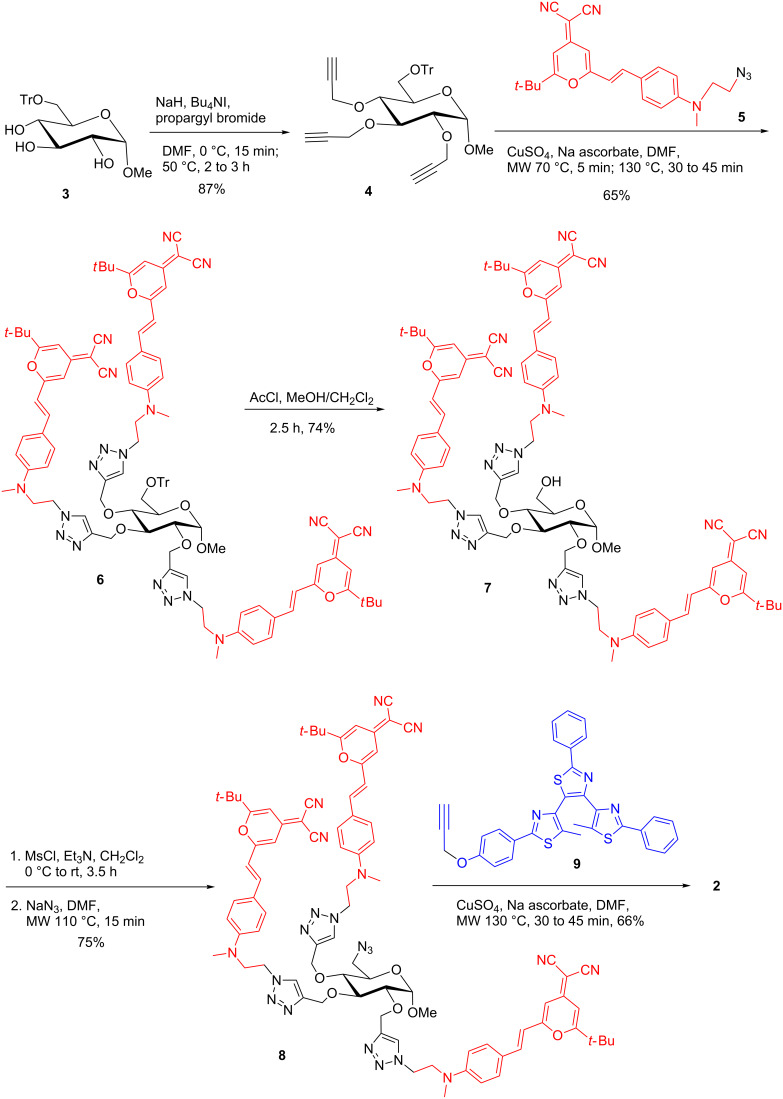
Synthesis of multichromophoric glucopyranoside **2**.

Synthesis of organic nano-architectures with a high degree of structural order and defined properties is challenging because sophisticated multistep experimental procedures are often implicated. Recently, the Cu(I)-catalyzed alkyne–azide cycloaddition reaction (CuAAC, an excellent example of click chemistry) has been demonstrated as a robust and highly efficient ligation tool to conjugate various azido- and alkyne-functionalized moieties [18–20]. Monosaccharides can be readily functionalized with several propargyl groups to synthesize, using click chemistry, multivalent neoglycoconjugates for recognition studies with carbohydrate-binding proteins (lectins) [21–24] or to develop light-harvesting antenna systems [17]. In order to introduce three DCM fluorophores and one photochromic species into the glycopyranoside scaffold, methyl 6-*O*-trityl-α-D-glucopyranoside **3** was chosen as starting material (Scheme 1). *O*-Propargylation followed by microwave-assisted CuAAC with azido-functionalized DCM fluorophore **5** [25] in the presence of copper sulfate and sodium ascorbate led to the fluorescent glucoside **6**. The trityl group was then removed by a catalytic amount of acetyl chloride in a mixture of MeOH and CH_2_Cl_2_. Subsequent activation as mesylated followed by “microwave-assisted” nucleophilic substitution with sodium azide afforded the corresponding 6-azido sugar **8** which was treated with alkyne-functionalized photochromic diarylethene **9** [26] to furnish the target compound **2** in 68% yield. The structure of this compound has been confirmed by NMR and HRMS spectra.

### Photophysical studies

**Figure 3:**
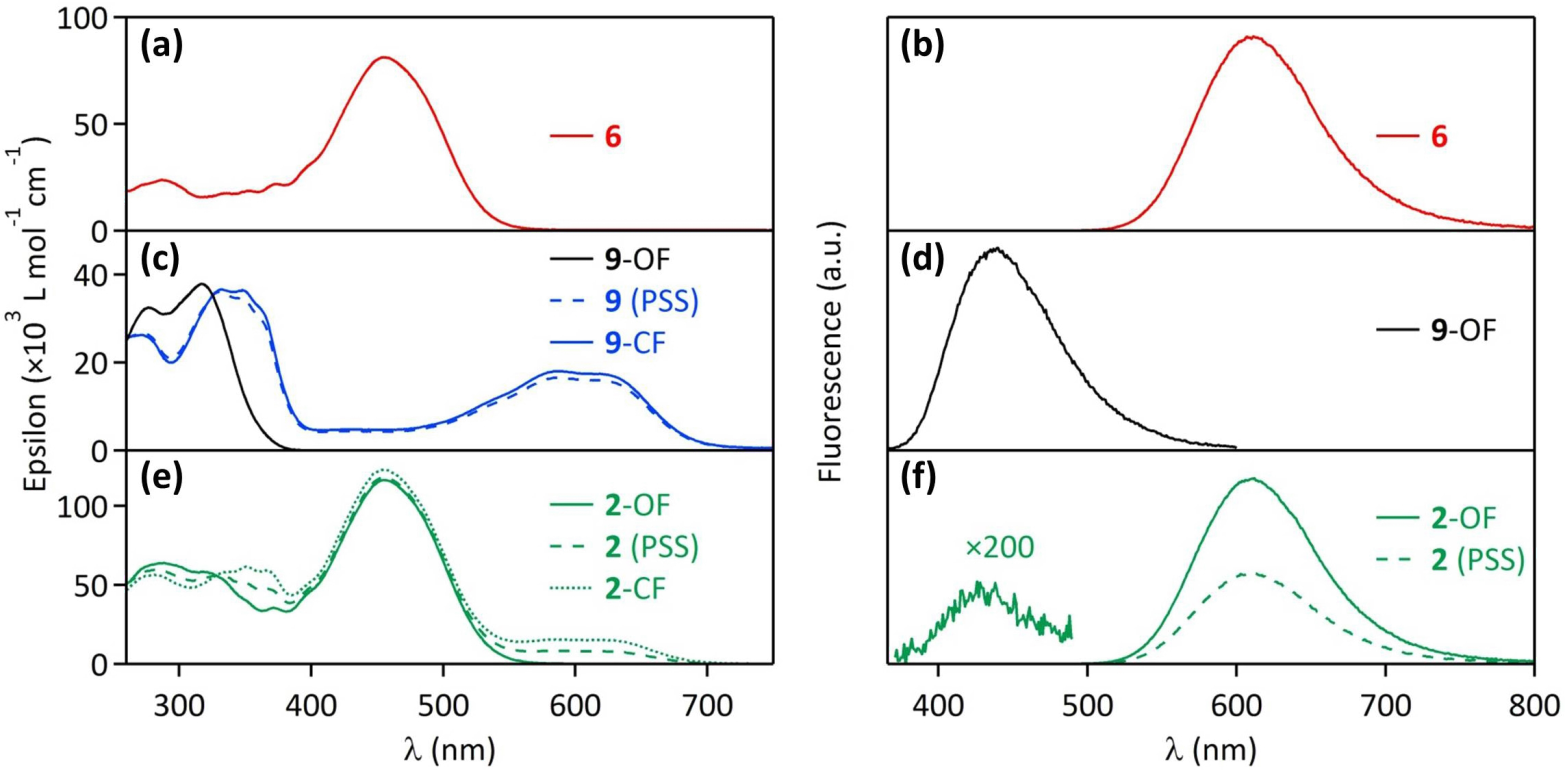
Absorption and fluorescence spectra of compounds **6**, **9**, and **2** in CH_3_CN: (a) absorption spectrum of **6**; (b) emission spectrum of **6** (λ_exc_ = 450 nm); (c) absorption spectra of **9**-OF, **9** at the photostationary state (PSS) under 335 nm illumination, isolated **9**-CF; (d) emission spectrum of **9**-OF (λ_exc_ = 325 nm); (e) absorption spectra of **2**-OF, **2** at the photostationary state (PSS) under 335 nm illumination (no noticeable change has been observed over 5 days, indicating the thermal stability of **2**), **2**-CF (obtained by extrapolation of the ^1^H NMR vs UV–visible absorption experiment, see text for details); (f) emission spectra of **2**-OF and **2** at the photostationary state (PSS) under 335 nm illumination (λ_exc_ = 325 nm).

**Figure 4:**
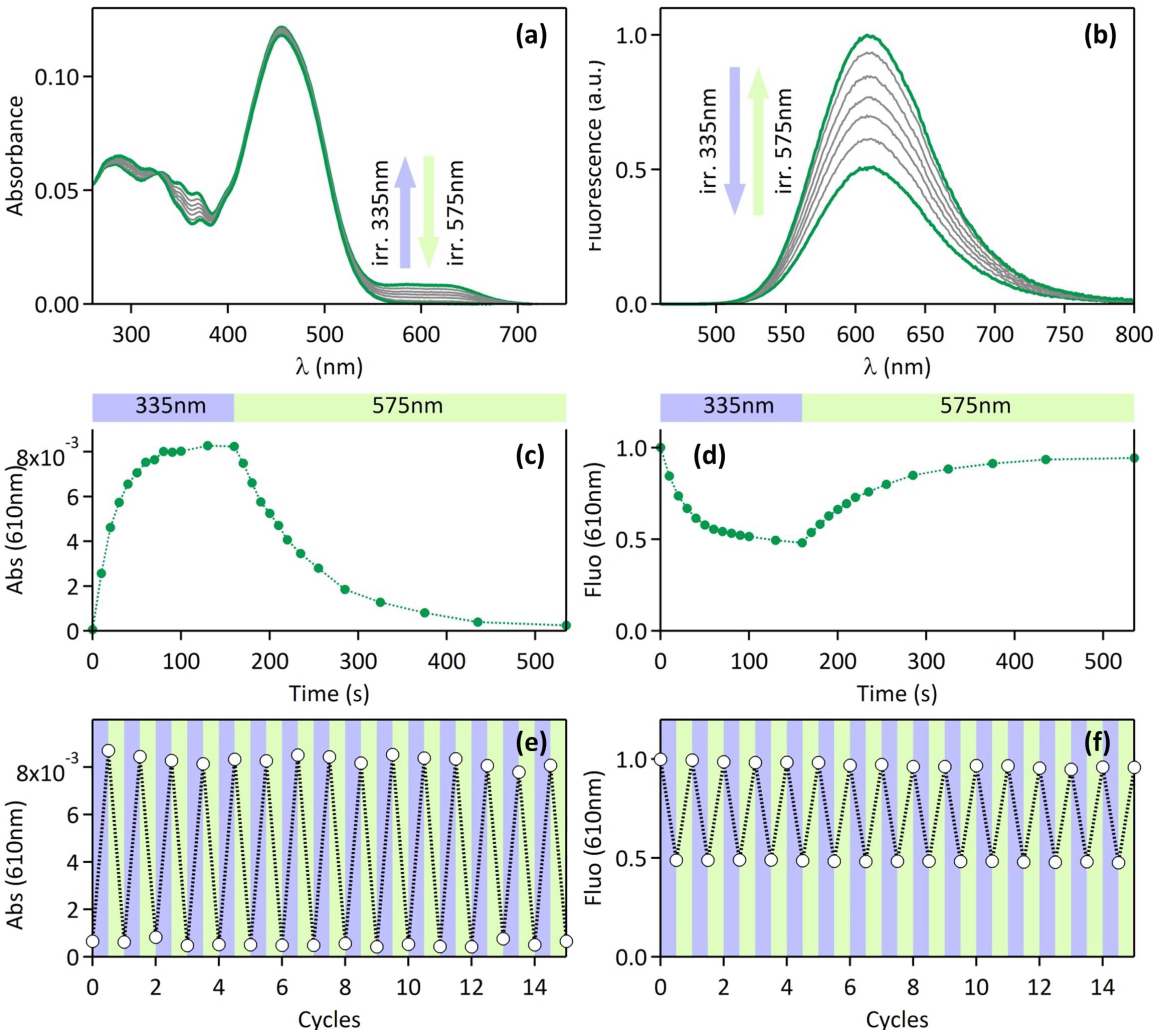
Absorption and fluorescence changes of compound **2** (1.0 μM in CH_3_CN) upon UV–visible irradiation: (a) absorption and (b) emission spectra (λ_exc_ = 450 nm) under increasing irradiation times at 335 nm; (c) time-evolution profile of absorption and (d) emission upon irradiation at 335 nm and 575 nm; (e) fatigue resistance followed by the absorption band at 610 nm and (f) the emission band at 610 nm under alternate 355 nm/575 nm irradiation cycles. Irradiation conditions at 335 nm: (a–d) 2.7 mW cm^−2^ and (e,f) 40 s at 7.2 mW cm^−2^. Irradiation conditions at 575 nm: (a–d) 3.1 mW cm^−2^ and (e,f) 120 s at 9.5 mW cm^−2^.

The fluorescent glucoside derivative **6**, bearing three DCM fluorophores, is used as a model fluorescent compound. Its absorption spectrum in acetonitrile, plotted in Figure 3a, shows a main large band peaking at 455 nm. It exhibits a wide fluorescence spectrum between 550 nm and 700 nm, with a maximum located at 610 nm (Figure 3b), associated with a fluorescence quantum yield Φ_F_ = 0.12. These spectroscopic characteristics are rather comparable to the absorption and fluorescence features of the fluorophore **5** [4]. The photophysical properties of the photochromic model compound **9** have been described in a previous report [4]. Briefly, the diarylethene **9** is originally in its colorless open form (**9**-OF), with an absorption spectrum in acetonitrile located in the UV range (λ_max_ = 317 nm, Figure 3c, full black line). In this state, **9**-OF is moderately fluorescent in the blue region (λ_max_ = 440 nm, Φ_F_ = 0.016, Figure 3d). Under irradiation at 335 nm, **9**-OF undergoes a cyclisation photoreaction yielding the molecule in its colored and non-fluorescent closed form (**9**-CF), as revealed by the appearance of a large absorption band peaking in the 500–700 nm range (Figure 3c, full blue line). The photostationary state (PSS) reached under 335 nm illumination is composed of 91% of **9**-CF and 9% of **9**-OF (Figure 3c, dashed line). The target compound **2** can be considered as the assembly of the fluorescent model derivative **6** and the photochromic model compound **9**. Indeed, before any UV irradiation, the absorption spectrum of **2** in its open form (**2**-OF) represents the overlay of the absorption feature of **6** and **9**-OF: as displayed in Figure 3e (full line), the absorption band at 455 nm matches to the three fluorophores, whereas the absorption bands at 286 nm and 325 nm correspond mostly to the photochromic diarylethene moiety. The fluorescence spectrum of **2**-OF shows a very weak blue emission band between 400 nm and 500 nm from the diarylethene unit, and a rather strong red emission band peaking at 610 nm from the three DCM dyes (Figure 3f). This dual emission will be discussed further, by a careful analysis of excitation spectra (vide infra). Figure 4a and b show that upon increasing irradiation times at 335 nm, an absorption band at 600 nm emerges, corresponding to the photochromic moiety in its thermally stable closed form, and the fluorescence emission is concomitantly quenched by 51%. As displayed in Figure 3, the emission band of the fluorophores in the 550–700 nm range overlaps well the absorption band of the photochromic derivative in its closed form, in the same spectral region. Therefore, the fluorescence quenching observed for the compound **2** under UV irradiation can be easily interpreted as the consequence of a FRET process from the DCM fluorophores (donors) to the diarylethene derivative in its colored closed form (acceptor), which plays the role of the quencher. The multichromophoric system **2** is completely reversible: under irradiation at 575 nm, the cycloreversion reaction **2**-CF → **2**-OF occurs, the absorption band centered at 610 nm drops back to zero and the fluorescence of the sample is fully recovered (Figure 4c and d). As shown in Figure 4e and f, several UV–visible irradiation cycles were applied to the system without any degradation of its photophysical properties, revealing its excellent fatigue resistance.

**Figure 5:**
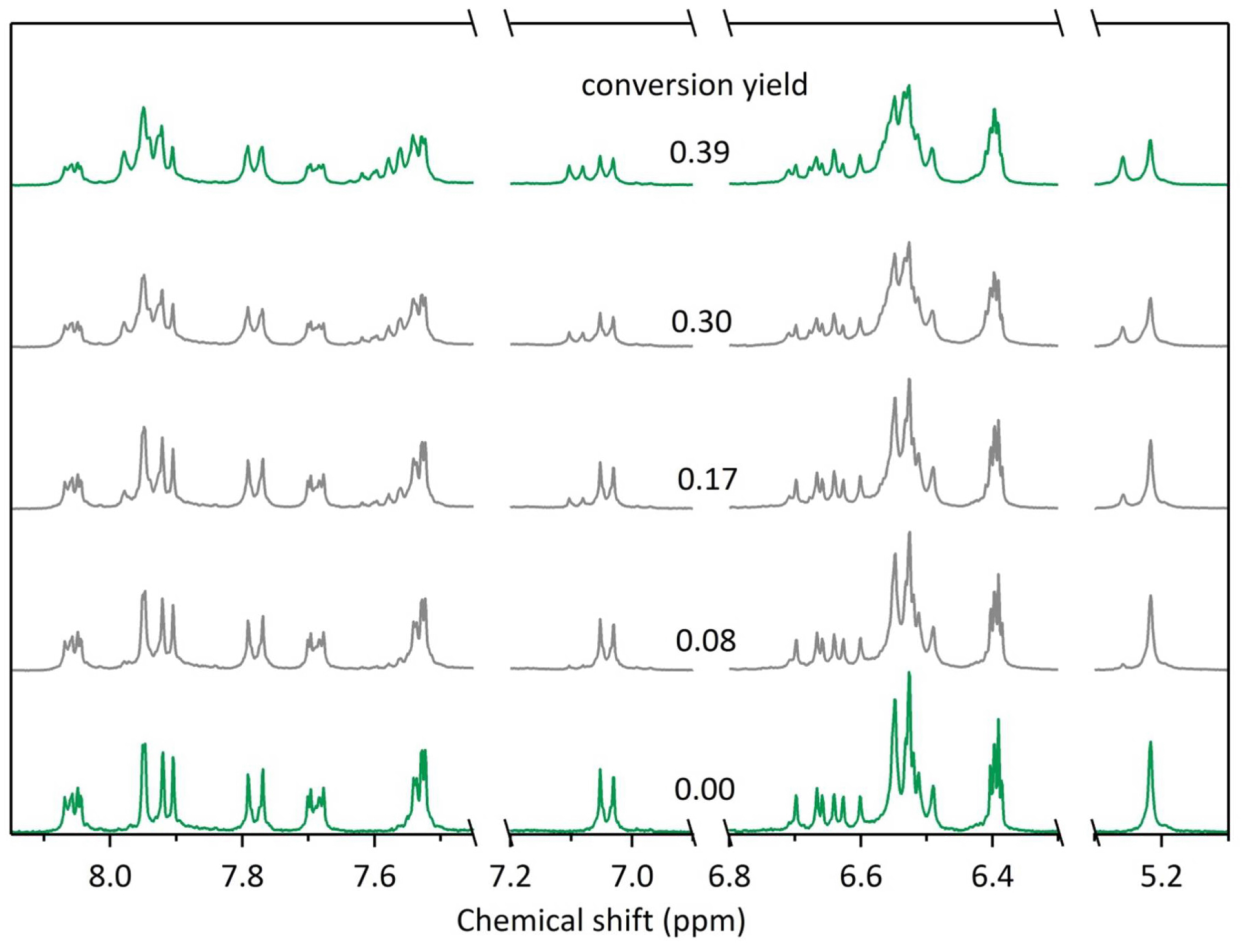
Partial ^1^H NMR spectra of compound **2** (11 μM in CD_3_CN/DMSO-*d*_6_ 4:1) before and after increasing irradiation times at 335 nm (from bottom to top). The conversion yield at each irradiation time is deduced from the integration of signals around 5.2–5.3 and 7.0–7.1 ppm.

^1^H NMR spectra were recorded under increasing irradiation times at 335 nm in order to follow the photoisomerisation of compound **2** (Figure 5), and corresponding UV–visible absorption spectra were measured, in order to correlate the absorption changes with the OF → CF conversion yield. Under 335 nm illumination, new signals appear near 5.2 ppm (for OCH_2_ group) and 6.5 to 8.0 ppm which are induced by the photocyclisation of the photochromic moiety from the open to the closed form. Due to higher concentration of NMR sample, the maximum conversion reached was about 39%. Such combined ^1^H NMR vs UV–visible absorption data allowed us to extrapolate the absorption spectrum of the dyad molecule in its pure closed form **2**-CF, as plotted in Figure 3e (dotted line). It appears that the PSS obtained for the compound **2** with a light-irradiation at 335 nm corresponds to a mixture of 53% of **2**-CF and 47% of **2**-OF. Such a conversion yield has to be compared to the fluorescence quenching of **2** observed after irradiation at 335 nm (51% fluorescence quenching, vide supra). Therefore, the incomplete fluorescence quenching is due to a limited photochromic conversion yield under UV light. Furthermore, since the photochromic conversion yield (53%) corresponds almost to the fluorescence quenching (51%), the FRET process appears to be extremely efficient: under irradiation at 335 nm, half of the multichromophoric system is still in its initial open form **2**-OF, associated with a strong fluorescence emission, and another half are promoted in their closed form **2**-CF, whose fluorescence is almost totally quenched by FRET.

**Figure 6:**
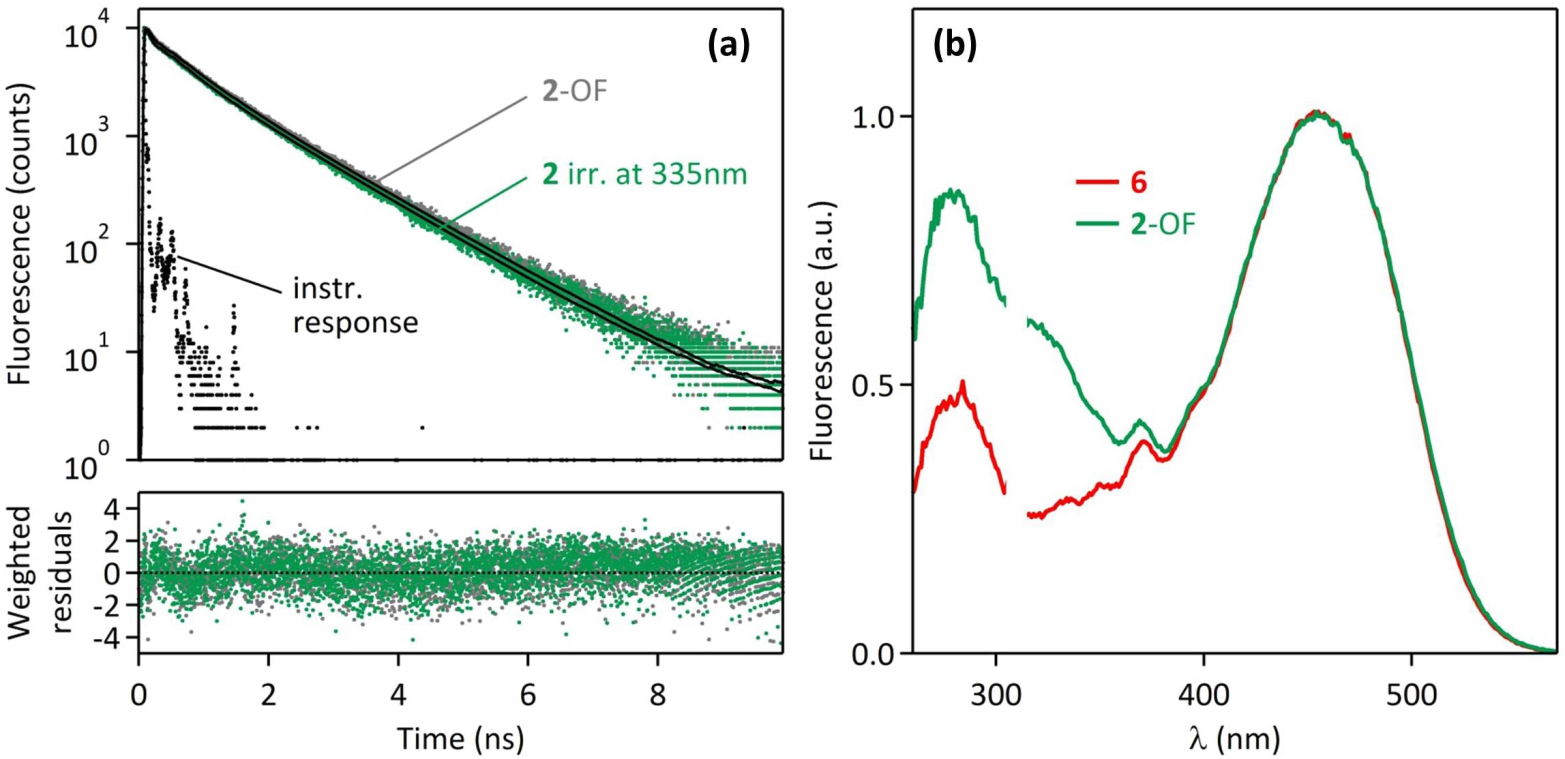
(a) Fluorescence decays (λ_exc_ = 475 nm, λ_em_ = 610 nm) of compound **2**-OF, and **2** after irradiation at 335 nm in CH_3_CN (7 mW cm^−2^). (b) Normalized excitation spectra (λ_em_ = 620 nm) of **6** (red line) and **2**-OF (green line) in CH_3_CN.

This phenomenon is well-supported by time-resolved fluorescence measurements. Fluorescence decay curves of **6**, **2**-OF, and **2** after irradiation at 335 nm were recorded in acetonitrile by the time-correlated single photon counting method (TCSPC) at λ_exc_ = 475 nm, and analyzed by a sum of three exponential components (Table 1). The model fluorescent glucoside derivative **6** shows a main time-constant at τ_1_ = 1.00 ns, a second component at τ_2_ = 0.35 ns, and a minor time-constant at τ_3_ = 0.04 ns. The latter one has been neglected in the following discussion, since it is very close to the time-resolution of our instrument, and associated with a very low fraction of intensity (0.02). The two other time-constants are attributed to the *E*-isomer (τ_1_) and the *Z*-isomer (τ_2_) of the fluorophores, which is consistent with our previous studies [4]. The fluorescence decays of **2**-OF and **2** after irradiation at 335 nm are plotted in Figure 6a, and the result of a global three-exponential fitting procedure is displayed on Table 1. The three time-constants obtained by this method are in the same range as the ones determined for **6**: τ_1_ = 1.26 ns, τ_2_ = 0.47 ns, τ_3_ = 0.05 ns, associated with the fraction of the intensities comparable to **6**. In a similar manner, the two significant contributions correspond to the *E* and *Z*-isomers of the fluorophores, respectively. Interestingly, the fluorescence decay curves of **2** before and after UV irradiation are overlapping, and the results of the fitting are identical in both cases, despite the fact that the fluorescence intensity is decreased by a factor of two (51% fluorescence quenching, vide supra). Such an observation is compatible with a static FRET quenching process. Indeed, when the PSS is reached under 335 nm irradiation, the population of **2**-OF molecules represents 47% of the whole system, behaves as the initial non-irradiated molecules and contributes to the fluorescence decay, whereas the population of **2**-CF molecules (53% of the system) is fully quenched through a very efficient FRET process, its contribution to the emission signal is negligible, and its decay-time is obviously below the time-resolution of our instrument. As a conclusion, the FRET process from the fluorophores to the closed form of the photochromic diarylethene is close to 100% in the molecule **2**.

**Table 1:** Fluorescence decay parameters of compounds **6** and **2** in CH_3_CN.

	τ_1_/ns (*a*_1_, *f*_1_^a^)	τ_2_/ns (*a*_2_, *f*_2_^a^)	τ_3_/ns (*a*_3_, *f*_3_^a^)	χ*^2^_R_*

**6**	1.00 (0.43, 0.79)	0.35 (0.29, 0.19)	0.04 (0.28, 0.02)	1.08
**2**-OF^b^	1.26 (0.40, 0.75)	0.47 (0.33, 0.23)	0.05 (0.27, 0.02)	1.16
**2** (PSS under irradiation at 335 nm)^b^	1.26 (0.35, 0.74)	0.47 (0.30, 0.23)	0.05 (0.35, 0.03)	1.12

^a^The fraction of intensities *f_i_* is defined as follows: *f*_i_ = *a*_i_τ_i_/Σ*a*_j_τ_j_. ^b^Results obtained by means of a global fitting procedure.

Fluorescence excitation spectra provide another evidence of FRET intramolecular phenomenon within the compound **2**. Indeed, the excitation spectrum of the model fluorescent glucoside **6**, displayed in Figure 6b (red curve), resembles to the shape of its absorption spectrum, with a large band centered at 455 nm. However, the excitation spectrum of the molecule **2**-OF recorded at λ_em_ = 620 nm (corresponding to the fluorophore emission) shows an additional contribution in the 250–350 nm range, which corresponds to the open form of the photochromic moiety, revealing a FRET process from the open form of the diarylethene unit (donor) to the DCM fluorophores (acceptors). This energy transfer pathway is actually allowed by the favorable spectral overlap in the 400–500 nm range between the blue emission of the photochromic moiety in its open form (see Figure 3d) and the absorption of the fluorophores (see Figure 3a). As evidenced previously on **1** [4], this "reverse" FRET explains why the emission of the photochromic moiety is almost absent in the fluorescence spectrum of the dyad **2**-OF (Figure 3f).

Finally, the limited conversion yield of the molecule **2** under irradiation in the UV (53%), compared to the model photochromic compound **9** (conversion yield = 91%) can be explained by this efficient double FRET effect: (i) the energy transfer from the open form of the photochromic unit to the fluorophores tends to deactivate the photochromic activity of **2**-OF, and (ii) the energy transfer from the fluorophores to the closed form of the photochromic unit contributes to favor the CF → OF cycloreversion reaction. Consequently, the PSS of **2** when irradiated at 335 nm is much lower than the photochromic moiety **9** alone, because of the highly efficient FRET processes.

## Conclusion

Through click chemistry, we have successfully introduced three DCM fluorophores and a DAE photochrome on the methyl α-D-glucopyranoside in good yield. The multichromophoric compound **2** can be reversibly switched upon UV and visible irradiation, and showed an excellent fatigue resistance. Under UV irradiation at 335 nm, the fluorescence was decreased by 51%. This value corresponds to the photochromic conversion yield at this photostationary state, with 53% of the DAE molecules promoted to their closed form. We therefore demonstrated that, at the molecular level, one single DAE moiety in the closed form induces the full fluorescence quenching of all three DCM moieties, by a FRET yield close to unity. This study is supported by time-resolved fluorescence experiments. This result represents a step forward, compared to our previous report on the 1/1 system **1**. The increase of the OF to CF conversion extent of the photochromic unit is among our future perspectives, since this could lead to a better ON vs OFF fluorescence contrast. By a careful molecular engineering, combining photophysical analysis and synthetic work, we are endeavoring to tune optimally the fluorophore/photochrome ratio. Glycosides, and more generally sugar molecules, may further provide appropriate "platforms".

## Experimental

### General details

Commercially available solvents and reagents were used without further purification. Compounds **3** [27], **5** [25] and **9** [26] were prepared according to the literature. Melting points were measured on a Kofler bench. Optical rotations were measured using a JASCO P-2000 polarimeter. Column chromatography was performed on Carlo Erba Silica Gel 60A (40–63 µm). Analytical thin-layer chromatography was performed on E. Merck aluminum percolated plates of Silica Gel 60F-254 with detection by UV. ^1^H and ^13^C NMR spectra were recorded on a Jeol ECS-400 spectrometer. HRMS–ESI spectra were recorded on a Bruker microTOF-Q II spectrometer or Bruker maXis using standard conditions.

Absorption spectra were recorded on a Cary-5000 spectrophotometer from Agilent Technologies. Corrected emission spectra were performed on a Fluorolog FL3-221 spectrofluorometer from Horiba Jobin-Yvon. The fluorescence quantum yields were determined by using quinine sulfate dihydrate in sulfuric acid (0.5 N) as a standard at λ_exc_ = 325 nm (Φ_F_ = 0.546) and coumarin 540A in ethanol as a standard at λ_exc_ = 450 nm (Φ_F_ = 0.544). Photochromic reactions were induced in situ by a continuous irradiation Hg–Xe lamp (Hamamatsu, LC6 Lightningcure, 200 W) equipped with narrow band interference ﬁlters of appropriate wavelengths (Semrock FF01-335/7-25 for λ_irr_ = 335 nm; FF01-575/25-25 for λ_irr_ = 575 nm). The irradiation power was measured using a photodiode from Ophir (PD300-UV). The photoconversion was followed by a combination of ^1^H NMR and UV–visible absorption spectra, realized by successive irradiation at 335 nm for a total time of 20 min. Fluorescence intensity decays were obtained by the time-correlated single-photon counting (TCSPC) method with femtosecond laser excitation using a set-up composed of a Ti:Sa laser (Tsunami, Spectra-Physics) pumped by a doubled Nd:YAG cw-laser (Millennia, Spectra-Physics), pumped itself by two laser diode arrays. Light pulses at 950 nm were selected by optoacoustic crystals at a repetition rate of 4 MHz, and then doubled at 475 nm by non-linear crystals. Fluorescence photons were detected through a band-pass filter 370–500 nm (a monochromator set at 620 nm, respectively) by means of a Hamamatsu MCP R3809U photomultiplier, connected to a constant-fraction discriminator. The time-to-amplitude converter was purchased from Tennelec. In the present investigation, the channel width was set to 3.1 ps. The instrumental response function was recorded before each decay measurement with a fwhm (full width at half-maximum) of ~25 ps. The fluorescence data were analyzed using the Globals software package developed at the Laboratory for Fluorescence Dynamics at the University of Illinois at Urbana-Champaign, which includes reconvolution analysis and non-linear least-squares minimization method. The shortest fluorescence decay time accessible by our instrumental set-up and our data analysis method was estimated to be around 10 ps (time-resolution).

### Methyl 2,3,4-tri-*O*-propargyl-6-*O*-trityl-α-D-glucopyranoside (**4**)

To a stirred solution of methyl 6-*O*-trityl-α-D-glucopyranoside (**3**, 3.08 g, 7.05 mmol) in distilled DMF (60 mL) under argon in an ice bath, were added Bu_4_NI (3.07 g, 8.32 mmol) and propargyl bromide (80% solution in toluene, 2.3 mL, 20.65 mmol). The NaH (60% in mineral oil, 1.02 g, 25.5 mmol) was then added slowly by portions. After 15 min, the mixture was heated at 50 °C over 2 to 3 h (brown to black color). After evaporation under vacuum, the residue was partitioned in a mixture of EtOAc/H_2_O (200:200 mL) and the aqueous layer was extracted with EtOAc (2 × 100 mL). The organic layers were combined, washed with brine, dried over MgSO_4_ and evaporated under vacuum to give the crude product which was purified by column chromatogrphy using a mixture of petroleum ether (PE):EtOAc (95:5, 9:1, 8:2) to give 87% of the desired compound (3.36 g, 6.10 mmol) as white solid, mp 96–97 °C; *R*_f_ = 0.64 (PE/EtOAc = 4:1); [α]_D_ +51 (*c* 0.5, CHCl_3_); ^1^H NMR (400 MHz, CDCl_3_) δ 2.23 (t, *J* = 2.3 Hz, 1H, CH≡), 2.45 (t, *J* = 2.3 Hz, 1H, CH≡), 2.50 (t, *J* = 2.3 Hz, 1H, CH≡), 3.12 (dd, *J* = 5.0, 10.1 Hz, 1H, H-6), 3.46 (s, 3H, OMe), 3.45–3.49 (m, 1H, H-6’), 3.53 (t, *J* = 9.2 Hz, 1H, H-4), 3.68 (dd, *J* = 3.7, 9.6 Hz, 1H, H-2), 3.69–3.72 (m, 1H, H-5), 3.78 (t, *J* = 9.2 Hz, 1H, H-3), 4.08 (dd, *J* = 2.8, 15.1 Hz, 1H, OCH), 4.25 (dd, *J* = 2.8, 15.1 Hz, 1H, OCH), 4.40–4.45 (m, 4H, 2×OCH_2_), 4.97 (d, *J* = 3.7 Hz, 1H, H-1), 7.21–7.32 (m, 9H, H_Ar_), 7.47–7.49 (m, 6H, H_Ar_) ppm; ^13^C NMR (100 MHz, CDCl_3_) δ 55.05 (OMe), 58.83, 59.94, 60.59 (OCH_2_), 62.83 (C-6), 69.80 (C-5), 74.31, 75.06 (C_q_), 77.36 (C-4), 79.46 (C-2), 79.87, 79.99, 80.19 (CH≡), 81.75 (C-3), 86.49 (C_q_), 97.79 (C-1), 127.11, 127.95, 128.86 (CH_Ar_), 144.11 (C_q_) ppm; HRMS–ESI (*m*/*z*): [M + Na]^+^ calcd for 573.2253; found: 573.2244.

### DCM-functionalized methyl 6-*O*-trityl-α-D-glucopyranoside **6**

To a solution of **4** (1.01 g, 1.83 mmol) in distilled DMF (15 mL), were added the azido-DCM **5** (2.23 g, 5.57 mmol), CuSO_4_ (110.9 mg, 0.44 mmol) and Na ascorbate (188 mg, 0.95 mmol). The reaction mixture was stirred at 70 °C during 5 min then at 130 °C during 30 to 45 min under microwave irradiation (700 rpm, monitoring by TLC), and poured into distilled water after cooling to room temperature. The precipitate was then filtred through a cellulose acetate filter (porosity 2 µm) under vacuum and washed with water, then purified by column chromatography using pure EtOAc, then EtOAc/acetone (gradient 9:1, 8:2, 5:5) then EtOAc/EtOH (7:3) to give 65% of the desired compound (2.09 g, 1.19 mol) as a red solid; mp 164–166 °C; *R*_f_ = 0.29 (EtOAc/acetone = 9:1); [α]_D_ +12 (*c* 0.5, CHCl_3_); ^1^H NMR (400 MHz, acetone-*d*_6_) δ 1.38 (s, 27H, H*_t-_*_Bu_), 2.84 (s, 3H, NMe), 2.85 (s, 3H, NMe), 2.85 (s, 3H, NMe), 3.10 (dd, *J* = 4.6, 10.1 Hz, 1H, H-6), 3.39–3.40 (m, 1H, H-6’), 3.41 (s, 3H, OMe), 3.50 (t, *J* = 9.5 Hz, 1H, OCH), 3.55 (dd, *J* = 3.7, 9.6 Hz, H-2), 3.62–3.70 (m, 2H, 2×OCH), 3.82–3.99 (m, 6H, 3×NCH_2_), 4.34 (d, *J* = 11.4 Hz, 1H, NCH), 4.58–4.86 (m, 11H, NCH, 2×NCH_2,_ 3×OCH_2_), 4.98 (d, *J* = 3.7 Hz, 1H, H-1), 6.45–6.46 (m, 3H, 3×CH=), 6.56–6.73 (m, 9H, 9×CH=), 6.81 (d, *J* = 16.0 Hz, 1H, CH=), 6.86 (d, *J* = 16.0 Hz, 1H, CH=), 6.87 (d, *J* = 16.0 Hz, 1H, CH=), 7.21–7.33 (m, 10H, 10×CH=), 7.39 (s, 1H, H_Triazole_), 7.41–7.53 (m, 14H, 14×CH=), 8.15 (s, 1H, H_Triazole_), 8.17 (s, 1H, H_Triazole_) ppm; ^13^C NMR (100 MHz, acetone-*d*_6_) δ 28.14 (Me*_t-_*_Bu_), 37.26 (C_q_), 38.63, 38.78 (NMe), 47.99, 48.12, 48.18, 52.89 (NCH_2_), 55.14 (OMe), 57.61 (C_q_), 63.50 (C-6), 64.41, 66.39, 66.98 (OCH_2_), 69.68 (C_q_), 70.96 (C-5), 78.46, 80.40, 81.91 (C-2,3,4), 87.03 (C_q_), 98.37 (C-1), 102.62, 106.02, 106.07, 112.67, 112.75, 114.21, 114.33 (CH=), 116.13, 116.18, 123.98, 124.07 (C_q_), 124.81, 125.29, 125.45 (CH_Triazole_), 127.86, 128.66, 129.56, 130.79, 130.84, 139.11, 139.22 (CH=), 145.00, 145.56, 145.91, 146.14, 151.13, 151.18, 157.49, 157.49, 161.50, 161.55, 173.02 (C_q_) ppm; HRMS–ESI (*m*/*z*): [M + H]^+^ calcd for 1751.8463; found: 1751.8397.

### DCM-functionalized methyl α-D-glucopyranoside **7**

To a stirred solution of compound **6** (940 mg, 0.54 mmol) in a mixture of CH_2_Cl_2_/MeOH (10/10 mL) cooled in an ice bath, was added acetyl chloride (115 µL, 1.61 mmol). After 2.5 h, the reaction was quenched by addition of a saturated NaHCO_3_ solution (5 mL). The mixture was extracted with CH_2_Cl_2_. The organic layers were combined, washed with brine, dried over MgSO_4_ and evaporated under vacuum. The product was purified by column chromatography using pure EtOAc followed by a mixture of EtOAc/acetone (gradient 9:1, 8:2, 7:3, 6:4) to give 74% of the desired compound (600 mg, 0.40 mmol) as a red solid; mp 156–158 °C; *R*_f_ = 0.22 (EtOAc/acetone = 4:1); [α]_D_ +25 (*c* 0.5, CHCl_3_); ^1^H NMR (400 MHz, CDCl_3_) δ 1.37 (s, 27H, H*_t-_*_Bu_), 2.89 (s, 3H, NMe), 2.91 (s, 3H, NMe), 2.92 (s, 3H, NMe), 3.37 (s, 3H, OMe), 3.47 (dd, *J* = 3.2, 9.6 Hz, 1H, H-2), 3.52–3.54 (m, 2H, H-3,4), 3.73 (s, 2H, H-6,6’), 3.80–3.83 (m, 1H, H-5), 3.92–3.94 (m, 6H, 3×NCH_2_), 4.58–4.60 (m, 6H, 3×NCH_2_), 4.74–4.97 (m, 7H, H-1, 3×OCH_2_), 6.45–6.65 (m, 16H, 16×CH=), 7.25–7.42 (m, 8H, 8×CH=), 7.63 (s, 1H, H_Triazole_), 7.87 (s, 1H, H_Triazole_), 8.00 (s, 1H, H_Triazole_) ppm; ^13^C NMR (100 MHz, CDCl_3_) δ 28.18 (Me*_t-_*_Bu_), 36.70 (C_q_), 38.59, 38.66, 38.74 (NMe), 47.49, 47.57, 52.53 (NCH_2_), 55.22 (OMe), 57.89, 57.94 (C_q_), 61.18 (C-6), 64.13, 65.75, 66.27 (OCH_2_), 69.60 (C_q_), 70.71, 77.36 (C-3,4), 79.39 (C-2), 81.42 (C-5), 97.51 (C-1), 102.43, 105.81, 111.98, 112.03, 113.81 (CH=), 115.70, 115.81, 115.86, 123.30, 123.35, 123.41 (C_q_), 123.86, 124.43, 124.74 (CH_Triazole_), 129.91, 137.95, 138.02 (CH=), 144.90, 145.32, 145.42, 149.84, 149.92, 149.98, 156.83, 160.01, 160.01, 160.05, 172.04 (C_q_) ppm. HRMS–ESI (*m*/*z*): [M + H]^+^ calcd for 1509.7367; found: 1509.7332; [M + 2H]^2+^ calcd for 755.3720; found: 755.3715.

### DCM-functionalized methyl 6-azido-6-deoxy-α-D-glucopyranoside **8**

To a stirred solution of compound **7** (414 mg, 0.27 mmol) in CH_2_Cl_2_ (2 mL) were added Et_3_N (125 µL, 0.90 mmol) and MsCl (52 µL, 0.67 mmol). After stirring 3.5 h, the mixture was treated with 10 mL of water and the aqueous layer was extracted with CH_2_Cl_2_ (3 × 20 mL). The organic layers were combined, washed with brine, dried over MgSO_4_ and evaporated under vacuum to a crude mesylate which was used without purification for the next step. To a solution of crude mesylate in DMF (3 mL) was added sodium azide (29.9 mg, 0.46 mmol) and the mixture was stirred at 110 °C during 15 min under microwave irradiation (700 rpm, monitoring by TLC). After cooling to room temperature, the reaction mixture was poured into 20 to 25 mL of distilled water. The precipitate was then filtred through cellulose acetate filter (porosity 2 µm) under vacuum and washed with water. The product was purified by column chromatography using EtOAc/acetone (gradient 1:0 to 9:1) to give 75% of the desired compound (328 mg, 0.21 mmol) as a red solid; mp 156–158 °C; *R*_f_ = 0.49 (EtOAc/acetone = 4:1); [α]_D_ +9 (*c* 0.5, CHCl_3_); ^1^H NMR (400 MHz, CDCl_3_) δ 1.37 (s, 27H, H*_t-_*_Bu_), 2.91 (s, 3H, NMe), 2.92 (s, 3H, NMe), 2.92 (s, 3H, NMe), 3.31–3.51 (m, 3H, H-4,6,6’), 3.39 (s, 3H, OMe), 3.49 (dd, *J* = 3.7, 9.6 Hz, 1H, H-2), 3.64–3.69 (m, 1H, H-5), 3.79 (t, *J* = 9.6 Hz, 1H, H-3), 3.90–3.97 (m, 6H, 3×NCH_2_), 4.57–4.64 (m, 6H, 3×NCH_2_), 4.65–4.97 (m, 7H, 3×OCH_2_, H-1), 6.46–6.66 (m, 16H, 16×CH=), 7.26–7.42 (m, 8H, 8×CH=), 7.69 (s, 1H, H_Triazole_), 7.89 (s, 1H, H_Triazole_), 8.00 (s, 1H, H_Triazole_) ppm; ^13^C NMR (100 MHz, CDCl_3_) δ 28.16 (Me*_t-_*_Bu_), 36.68 (C_q_), 38.54, 38.65, 38.71 (NMe), 47.51 (NCH_2_), 51.22 (C-6), 52.52 (NCH_2_), 55.39 (OMe), 57.84, 57.91, 57.91 (C_q_), 64.26, 65.87, 66.28 (OCH_2_), 69.57 (C_q_), 69.83 (C-5), 77.37 (C_q_), 78.00 (C-4), 79.29 (C-2), 81.08 (C-3), 97.43 (C-1), 102.41, 105.80, 111.95, 112.01, 113.73, 113.78 (CH=), 115.70, 115.83, 123.31, 123.37 (C_q_), 124.13, 124.43, 124.77 (CH_Triazole_), 129.89 (C_q_), 137.89, 137.93, 138.02 (CH=), 144.81, 144.86, 145.27, 149.86, 149.96, 149.96, 156.80, 159.97, 160.03, 172.02 (C_q_) ppm; HRMS–ESI (*m*/*z*): [M + H]^+^ calcd for 1534.7432; found: 1534.7381; [M + 2H]^2+^ calcd for 767.8753; found: 767.8746.

### DCM and DAE-functionalized methyl α-D-glucopyranoside **2**

To a solution of compound **8** (51.1 mg, 0.033 mmol) in DMF (2 mL) were added the photochromic compound **9** (62.0 mg, 0.111 mmol), CuSO_4_ (2.5 mg, 0.010 mmol) and Na ascorbate (6.8 mg, 0.034 mmol). The reaction mixture was stirred at 70 °C during 5 min then at 130 °C during 30 to 45 min under microwave irradiation (700 rpm, monitoring by TLC), and poured into distilled water after cooling to room temperature. The precipitate was then filtred through a cellulose acetate filter (porosity 2 µm) under vacuum and washed with water, then purified by column chromatogrphy using EtOAc:ethanol (9:1) to give 66% of the desired compound (45.8 mg, 0.022 mmol) as a red solid; mp 181–183 °C; *R*_f_ = 0.53 (EtOAc/acetone = 4:1); [α]_D_ +45 (*c* 0.5, CHCl_3_); ^1^H NMR (400 MHz, CDCl_3_) δ 1.36 (s, 27H, H*_t-_*_Bu_), 2.09 (s, 3H, Me), 2.54 (s, 3H, Me), 2.88 (s, 3H, NMe), 2.90 (s, 3H, NMe), 2.92 (s, 3H, NMe), 2.97 (t, *J* = 9.6 Hz, 1H, H-4), 3.21 (s, 3H, OMe), 3.35 (dd, *J* = 3.2, 9.6 Hz, 1H, H-2), 3.79–3.93 (m, 8H, H-3,5, 3×NCH_2_), 4.45–4.81 (m, 13H, H-1,6,6’, 3×NCH_2_, 2×OCH_2_), 4.93 (d, *J* = 11.4 Hz, 1H, OCH), 4.97 (d, *J* = 11.5 Hz, 1H, OCH), 5.26 (s, 2H, OCH_2_), 6.45–6.64 (m, 15H, 15×CH=), 7.02 (d, *J* = 8.7 Hz, 2H, 2×CH=), 7.26–7.49 (m, 15H, 15×CH=), 7.77–7.79 (m, 4H, 4×CH=), 7.88 (d, 2H, *J* = 8.7 Hz, 2×CH=), 7.97 (s, 1H, H_Triazole_), 8.03–8.09 (m, 3H, 3×CH=) ppm; ^13^C NMR (100 MHz, CDCl_3_) δ 12.46, 12.94 (Me), 28.25 (Me*_t-_*_Bu_), 36.77 (C_q_), 38.79, 38.88 (NMe), 47.56, 47.68 (NCH_2_), 50.54 (C-6), 52.56, 52.62, 52.72 (NCH_2_), 55.56 (OMe), 58.20, 58.28 (C_q_), 62.07, 64.23, 65.96, 66.27 (OCH_2_), 68.89 (CH), 69.65 (C_q_), 77.36, 79.31, 81.05 (OCH), 97.56 (C-1), 102.54, 112.21, 113.95, 114.05, 114.09, 115.29 (CH=), 115.71, 115.87, 123.51, 123.55, 123.60, 123.66 (C_q_), 124.57, 124.68, 124.94, 126.41, 126.72, 128.20, 128.89, 129.08, 129.95, 130.40 (CH=), 133.59, 133.68 (C_q_), 137.89, 137.95, 138.02 (CH=), 143.90, 144.78, 144.99, 145.06, 149.85, 156.89, 160.02, 163.93, 164.13, 167.30, 172.08 (C_q_) ppm; HRMS–ESI (*m*/*z*): [M + 2H]^2+^ calcd for 1048.4254; found: 1048.4247; [M + 3H]^3+^ calcd for 699.2860; found: 699.2845.

## References

[1] Irie M (2000). Photochromism: Memories and Switches. Chem Rev.

[2] Fukaminato T (2011). J Photochem Photobiol, C.

[3] Guo Z, Zhu W, Tian H (2012). Chem Commun.

[4] Ouhenia-Ouadahi K, Métivier R, Maisonneuve S, Jacquart A, Xie J, Léaustic A, Yu P, Nakatani K (2012). Photochem Photobiol Sci.

[5] Bossi M, Belov V, Polyakova S, Hell S W (2006). Angew Chem, Int Ed.

[6] Berberich M, Krause A-M, Orlandi M, Scandola F, Würthner F (2008). Angew Chem, Int Ed.

[7] Irie M, Fukaminato T, Sasaki T, Tamai N, Kawai T (2002). Nature.

[8] Jiang G, Wang S, Yan W, Jiang L, Song Y, Tian H, Zhu D (2006). Chem Mater.

[9] Golovkova T A, Kozlov D V, Neckers D C (2005). J Org Chem.

[10] Wu S, Luo Y, Zeng F, Chen J, Chen Y, Tong Z (2007). Angew Chem, Int Ed.

[11] Del Guerzo A, Olive A G L, Reichwagen J, Hopf H, Desvergne J-P (2005). J Am Chem Soc.

[12] Fölling J, Polyakova S, Belov V, van Blaaderen A, Bossi M L, Hell S W (2008). Small.

[13] Métivier R, Badré S, Méallet-Renault R, Yu P, Pansu R B, Nakatani K (2009). J Phys Chem C.

[14] Wu Y, Xie Y, Zhang Q, Tian H, Zhu W, Li A D Q (2014). Angew Chem, Int Ed.

[15] Nakashima T, Kajiki Y, Fukumoto S, Taguchi M, Nagao S, Hirota S, Kawai T (2012). J Am Chem Soc.

[16] Snegir S V, Marchenko A A, Yu P, Maurel F, Kapitanchuk O L, Mazerat S, Lepeltier M, Léaustic A, Lacaze E (2011). J Phys Chem Lett.

[17] Bonaccorsi P, Aversa M C, Barattucci A, Papalia T, Puntoriero F, Campagna S (2012). Chem Commun.

[18] Kolb H C, Finn M G, Sharpless K B (2001). Angew Chem, Int Ed.

[19] Tornøe C W, Christensen C, Meldal M (2002). J Org Chem.

[20] Wu P, Feldman A K, Nugent A K, Hawker C J, Scheel A, Voit B, Pyun J, Fréchet J M J, Sharpless K B, Fokin V V (2004). Angew Chem, Int Ed.

[21] Wu P, Chen X, Hu N, Tam U C, Blixt O, Zettl A, Bertozzi C R (2008). Angew Chem, Int Ed.

[22] Gao Y, Eguchi A, Kakehi K, Lee Y C (2005). Bioorg Med Chem.

[23] Perez-Balderas F, Morales-Sanfrutos J, Hernandez-Mateo F, Isac-García J, Santoyo-Gonzalez F (2009). Eur J Org Chem.

[24] Ortega-Muñoz M, Perez-Balderas F, Morales-Sanfrutos J, Hernandez-Mateo F, Isac-García J, Santoyo-Gonzalez F (2009). Eur J Org Chem.

[25] Yu Y, Bogliotti N, Maisonneuve S, Tang J, Xie J (2013). Tetrahedron Lett.

[26] Ouhenia-Ouadahi K, Yasukuni R, Yu P, Laurent G, Pavageau C, Grand J, Guérin J, Léaustic A, Félidj N, Aubard J (2014). Chem Commun.

[27] Collins D J, Hibberd A I, Skelton B W, White A H (1998). Aust J Chem.

